# The Plant Target of Rapamycin: A Conduc TOR of Nutrition and Metabolism in Photosynthetic Organisms

**DOI:** 10.3390/genes11111285

**Published:** 2020-10-29

**Authors:** Camille Ingargiola, Gustavo Turqueto Duarte, Christophe Robaglia, Anne-Sophie Leprince, Christian Meyer

**Affiliations:** 1 Institut Jean-Pierre Bourgin (IJPB), INRAE, AgroParisTech, Université Paris-Saclay, 78000 Versailles, France; camille.ingargiola@inrae.fr (C.I.); duarte.gst@gmail.com (G.T.D.); anne-sophie.leprince@inrae.fr (A.-S.L.); 2 Max Planck Institute of Molecular Plant Physiology, 14476 Potsdam, Germany; 3 Laboratoire de Génétique et Biophysique des Plantes, Faculté des Sciences de Luminy, UMR 7265, CEA, CNRS, BIAM, Aix Marseille Université, 13009 Marseille, France; christophe.robaglia@univ-amu.fr; 4 Faculté des Sciences et d’Ingénierie, Sorbonne Université, UFR 927, 4 Place Jussieu, 75252 Paris, France

**Keywords:** target of rapamycin (TOR), plant, metabolism, photosynthetic organisms, nutrition, sugars, carbon, nitrogen, sulfur

## Abstract

Living organisms possess many mechanisms to sense nutrients and favorable conditions, which allow them to grow and develop. Photosynthetic organisms are very diverse, from green unicellular algae to multicellular flowering plants, but most of them are sessile and thus unable to escape from the biotic and abiotic stresses they experience. The Target of Rapamycin (TOR) signaling pathway is conserved in all eukaryotes and acts as a central regulatory hub between growth and extrinsic factors, such as nutrients or stress. However, relatively little is known about the regulations and roles of this pathway in plants and algae. Although some features of the TOR pathway seem to have been highly conserved throughout evolution, others clearly differ in plants, perhaps reflecting adaptations to different lifestyles and the rewiring of this primordial signaling module to adapt to specific requirements. Indeed, TOR is involved in plant responses to a vast array of signals including nutrients, hormones, light, stresses or pathogens. In this review, we will summarize recent studies that address the regulations of TOR by nutrients in photosynthetic organisms, and the roles of TOR in controlling important metabolic pathways, highlighting similarities and differences with the other eukaryotes.

## 1. Introduction

As early as 1826, the French physiologist Henri Dutrochet stated that biology is one and that there is not a clear demarcation line between animal and plant physiology. Therefore, in these early days he thought that one could learn a lot from plant physiology when studying animals (and vice versa). He was indeed right, but the animal and plant field have diverged in their approaches when studying physiology and cell biology [1].

During their evolution from the Last Eukaryotic Common Ancestor (LECA), plants have diverged from animals and other eukaryotes in many ways. They acquired multicellularity and the ability to live outside water independently from animals but their most prominent feature is probably their ability to synthesize organic C molecules like sugars from light energy. This was made possible after the symbiotic acquisition of photosynthetic cyanobacteria that evolved into chloroplasts. Like yeasts, but unlike animals, plants are also able to synthesize organic N (for example amino acids, nucleotides or specialized metabolites) and S molecules from inorganic sources, which are found in their environment.

Finally, plants can produce new organs (for example leaves, roots or flowers) throughout their life due to continuous cell divisions in meristems that are located at the growing tips of roots and shoots, and from which most postembryonic structures are derived.

These characteristics allow plants to adapt to changing or adverse conditions from which they cannot escape. Nutrients availability, phytohormones, light intensity, pathogens, water or thermic stress are some of the numerous signals or constraints to which photosynthetic organisms are subjected. Therefore, plants and algae need to sense these different external or internal cues for adjusting their metabolism, growth and development to survive these sometimes harsh conditions (for a review see [2,3]).

In all eukaryotes since the LECA, conserved and ancient regulatory bricks allow the coupling of cell and tissue growth to the availability of nutrients and energy. These connections are essential to maintain cell homeostasis in between the assimilation and the use of nutrients, and must rely on efficient sensing mechanisms. One of the most central and conserved nutrient sensing pathway involves the Target of Rapamycin (TOR) kinase signaling cascade [4]. TOR is a central regulator of metabolism and growth processes in all eukaryotic cells, promoting anabolism in favorable conditions while inhibiting catabolism and protein degradation by autophagy and the proteasome pathway [5,6,7,8]. Furthermore, plant life depends on a close coupling of many environmental inputs (light and nutrients, but also abiotic or biotic stresses) with complex adaptive growth responses like root development or the switch from vegetative to reproductive growth. Given its roles in other eukaryotes, the TOR kinase appears as a good candidate to perform these connections between environmental cues and developmental processes.

In animals and yeast a wealth of studies have identified in the recent years multiple upstream regulators controlling TOR activity [6,9]. This interest in TOR was motivated by the fact that modifications of this kinase activity can cause cancers or metabolic diseases [5,10]. The TOR inducing or repressing factors include nutrients like sugars or amino acids, hormones like insulin or other signaling pathways.

The following review will emphasize how plant nutrients signaling affects the regulation of the TOR kinase and how TOR in turn controls many arms of plant metabolism. The plant and algae TOR signaling network has been recently reviewed in several excellent papers (for algae see [11,12]; for multicellular plants see for example [13,14,15,16,17]). Therefore, we will focus on the control of TOR activity in plants by nutrients, and the role of this kinase in regulating metabolic pathways, concentrating on C and N metabolisms.

## 2. The TOR Complex in Plants and Algae

**Figure 1:**
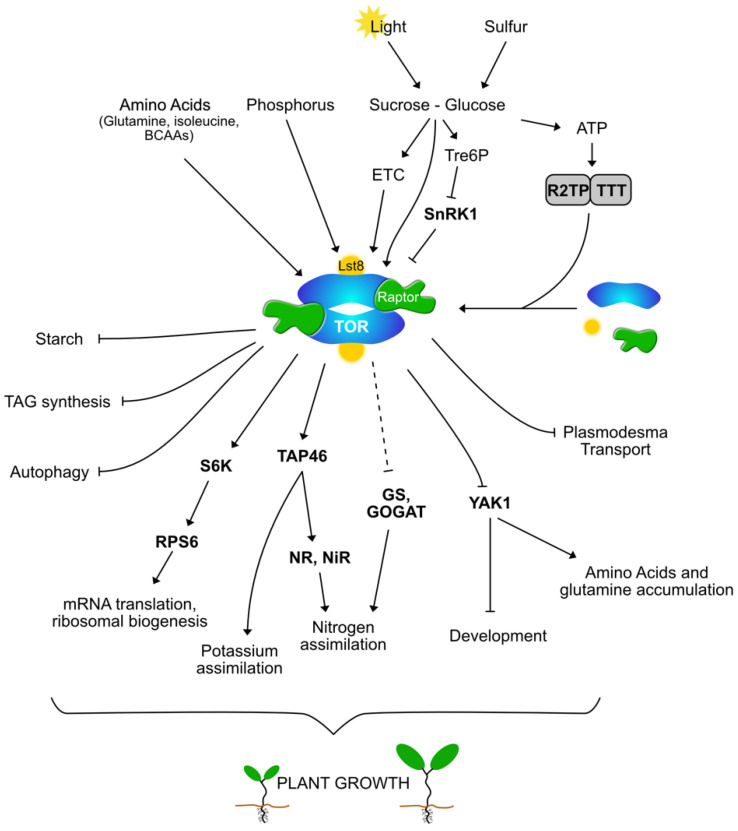
Regulations and outputs of the TOR signaling pathway in photosynthetic organisms: TOR is a conduc TOR of nutritional and metabolic processes. **Legend:** The TOR (Target of Rapamycin) protein kinase is a central regulatory hub connecting various environmental and internal signals with the plant and algal metabolic and growth processes. This kinase is the heart of the evolutionary conserved TORC1 complex in which it interacts with LST8 (Lethal with Sec Thirteen protein 8) and RAPTOR (Regulatory-Associated Protein of TOR). The association of R2TP (Pontin/RuvBL1-Reptin/RuvBL2-Spaghetti/Tah1-Pih1), TTT (Tel2-Tti1-Tti2) complex and HSP90 chaperone allow the dimerization and stabilization of the TORC1 complex depending on ATP levels. Light and photosynthesis produce sugars, which, through the ETC (Electron Transport Chain), are known to stimulate TOR activity and to inhibit SnRK1 (Snf1-Related Kinase 1), the antagonist kinase of TOR. SnRK1 is activated by low energy and nutrient conditions and phosphorylates RAPTOR, inhibiting TOR activity. Phosphorus, sulfur, nitrogen and amino acids also stimulate TOR activity. In response to these signals, TOR acts on different targets like S6K (ribosomal protein S6 kinase), YAK1 (Yet another Kinase 1) or TAP46 (PP2A regulatory subunit TAP46) to regulate mRNA translation, nutritional and metabolic processes and in fine plant growth.

TOR is a large Ser/Thr kinase protein, belonging to the Phosphatidylinositol Kinase-related Kinase (PIKK) family that also comprises of the conserved ATM and ATR kinases (see [18,19] for reviews). In yeast and mammals, the TOR kinase is involved in two different complexes, TORC1 and TORC2, which differ by the partners of the TOR kinase [5,6]. In plants and green algae, only the TORC1 complex seems to be present, where TOR interacts with two protein partners, RAPTOR and LST8, which are well conserved among eukaryotes [4]. TOR and other PIKKinases need the TTT (**T**el2-**T**ti1-**T**ti2) complex for their maturation and dimerization together with the R2TP (Pontin/**R**uvBL1-Reptin/**R**uvBL2-Spaghetti/**T**ah1-**P**ih1) complex and the HSP90 chaperone. Pontin-Reptin is an AAA+ ATPase-containing complex. In animals, it has been shown that deprivation of C and N nutrients lead to a decrease in ATP concentration and to an inhibition of the R2TP complex, which in turn will reduce the dimerization of the TORC1 complex and its kinase activity [20]. This mechanism thus provides a link between the sensing of energy supply and the regulation of the TORC1 activity. In the model plant *Arabidopsis*, all three components of the TTT complex were found to interact with the LST8 protein together with Reptin-Pontin homologs [21] (Figure 1). Brunkard et al. [22] identified in *Arabidopsis* a *reptin* mutant that displays a higher transport of molecules through plasmodesmata, pores that allow communication between adjacent plant cells, together with a decreased TOR activity. Accordingly, it was observed by the same authors that TOR repress transport of macromolecules through plasmodesmata in *Arabidopsis*. Lower TOR activity was found in *reptin*, but also *sphaghetti* and *telo2 Arabidopsis* mutants. Similarly, *tti2* (telo2 interacting protein2) mutants in maize have very reduced TOR activity [23].

So far, only the TORC1 protein partners were identified in plants, and many other components of the animal TOR signaling pathways seem to be missing [24]. In *Arabidopsis*, *tor* mutants are lethal at an early stage of development [25], indicating that the TOR kinase plays an essential role in the embryo development. The TOR partners RAPTOR and LST8 are both encoded by two genes in *Arabidopsis*, respectively *RAPTOR1*, *RAPTOR2* and *LST8-1*, *LST8-2* [26,27,28]. Mutations in single *RAPTOR* or *LST8* genes, and even in the two *RAPTOR* genes, are viable but the mutants display development defects and an altered TOR signaling [26,27,28,29,30]. Structural and interaction studies have shown that LST8 binds the TOR kinase domain [27,31]. This binding is necessary to stabilize and fully activate TOR. Indeed, TOR activity in *Arabidopsis lst8* mutants does not respond any longer to sugar activation [32]. It has been shown in animals and yeast that RAPTOR interacts with the HEAT repeats of TOR and presents substrates to the kinase domain [31,33]. In yeast, the KOG1 (RAPTOR in yeast) protein is involved in the oligomerization of inactive TORC1 complexes in hollow helices after glucose removal [34].

Contrary to yeast and mammals, *Arabidopsis* is relatively insensitive in most growth conditions to rapamycin, the first discovered TOR inhibitor [25,35]. However, rapamycin seems to inhibit growth of *Arabidopsis* plantlets [36] or cells [21] in liquid culture, maybe because hypoxia enhances the action of rapamycin in plants [37].

Other mTOR inhibitors like AZD-8055 (AZD) or TORIN2, which interfere with ATP binding, can inactivate TOR activity in plants [35,38]. Unlike plants, the unicellular green alga *Chlamydomonas* is sensitive to rapamycin, which has a strong effect on growth and metabolism [39,40].

Recently phosphoproteomic and interactomic analyses in *Arabidopsis* [21] or *Chlamydomonas* [40,41] have identified both plant-specific and conserved TOR targets and interactors. These studies provide further evidence that there is no clear phosphorylation consensus motif for the TOR kinase, except maybe for a Pro at position +1 and possibly a Gly at position −1 relative to the phosphorylated Ser/Thr residue. TOR is known to be a major regulator of the different steps of mRNA translation in eukaryotes [4,8]. Accordingly, many of the identified TOR targets are related to the control of translation, including LARP1 (La-Related Protein 1), components of the translation initiation complex and RPS6 (Ribosomal Protein S6). It was already known that *Arabidopsis* RPS6 was phosphorylated by S6 kinase (S6K) on C-terminal Ser residues [33] and RPS6 phosphorylation was later shown to be induced by sugar in a TOR-dependent manner after a phosphoproteomic analysis of the ribosomal fraction in *Arabidopsis* [42]. Interestingly, the decrease in C-terminal RPS6 phosphorylation is always among the most robust output of TOR inactivation, and this dephosphorylation is conserved among eukaryotes [21,40,43,44]. As a consequence, RPS6 phosphorylation is an excellent output for TOR activity in plants that can be monitored by a specific phosphoantibody like in other eukaryotes [42,43].

The dual specificity Tyr/Ser YAK1 kinase, an ortholog of the yeast YAK1 (Yet Another Kinase1) and of the animal DYRK1a, has recently been identified as a strong suppressor of the *lst8* mutation and as a direct TOR substrate in *Arabidopsis* [21,32]. Mutations in YAK1 were also identified in a screen for resistance to TOR inhibitors, and shown to control the SIAMESE-related cyclin-dependent kinase inhibitors [45]. These convergent results suggest that activation of YAK1 is a major contributor to the effects of TOR inhibition on growth and metabolism.

## 3. SnRK1 and TOR in Plants: An Intricate Reciprocal Interaction

The regulations and roles of TOR are less known in plants compared to yeast or animals. Generally, when the environmental conditions are favorable, the TORC1 complex is activated and stimulates anabolic reactions like mRNA translation, cellular growth and plant development (for a summary see [46] and Figure 1) and inhibits catabolic process like autophagy [47]. Moreover, TOR seems to participate to the responses to biotic and abiotic stresses like the regulation of cold acclimation [4]. Indeed, mutation of the *THADA* conserved gene involved in cold acclimation results in a low TOR activity [48]. In response to stress, TORC1 is often inhibited, which leads to stimulated autophagy [49]. In plants, this TOR inactivation partly depends on the activation of SnRK1 (Snf1-related kinase 1), an antagonist kinase of TOR [49,50]. SnRK1 is the ortholog of the AMP-activated kinase (AMPK) in animals and of the sucrose non-fermenting 1 kinase (Snf1) in yeast. SnRK1 interacts with the *Arabidopsis* TORC1 complex and inhibits its activity by phosphorylating the RAPTOR protein like in other eukaryotes [44]. In line with these findings, it was recently observed that the two SnRK1 catalytic subunits interact with the RAPTOR protein [21]. Furthermore, the reciprocal regulation was finally identified in fission yeast and animals. It was indeed recently shown that TOR phosphorylates a conserved residue on AMPK to inhibit its activity [51]. Interestingly this Ser residue is conserved in all eukaryotes including *Arabidopsis* (Ser283 in SnRK1/Kin11 catalytic subunit).

Even if plant components of both the SnRK1 and TOR complexes differ from the ones found in animals and yeast, the overall organization and interactions between these two mega complexes seem to have been conserved throughout evolution. A very recent paper describes a dual role for the SnRK2 kinases, plant specific members of the SnRK kinase family, in both inactivating SnRK1 in optimal growth conditions (and hence activating TOR) while activating SnRK1 in response to ABA [52]. Finally, it is tempting to hypothesize that all these complex kinase complexes could be tethered together to allow reciprocal and intertwined cross-regulations, which would limit their activation and maintain equilibrium within the cell.

## 4. The Sweet Side of TOR: Sugars as Regulatory Factors and Outputs

With the advent of methods allowing one to measure TOR activity in plants, it became possible to explore the regulations of this kinase by nutrient signals. These methods are the same as the ones used in the animal or yeast fields, and are mainly based on the detection of S6K activation by a TOR-dependent phosphorylation. This phosphorylation can be monitored directly by using a phospho-specific S6K antibody [36] or by a shift in electrophoretic mobility following phosphorylation of a HA-tagged S6K expressed in plants [21]. The S6K activation can also be detected by measuring RPS6 phosphorylation using a specific phospho-antibody [42,43]. The higher level of the ribosomal protein RPS6, together with the robustness, linearity and amplitude of its phosphorylation by TOR/S6K, facilitates the detection of phosphoRPS6 in plant extracts. Conversely, S6K is expressed at lower levels but has the advantage of being a direct TOR substrate.

It then became quickly obvious that, like in other eukaryotes, plant TOR activity is strongly and robustly induced by sugars (sucrose, glucose or fructose) and, consistently, highly repressed by sugar starvation [36,42,53]. In *lst8* mutants, TOR activity is reduced but also insensitive to the addition of sucrose to starved *Arabidopsis* seedlings. Surprisingly, the suppression by mutations of the YAK1 kinase of most of the phenotypic and metabolic defects in the *lst8* mutant does not restore induction of TOR activity by sugars [32]. When activated by sugars, TOR promotes the synthesis of proteins and nucleotides needed for cell growth and division [5,8,16]. Thus, TOR serves as an intermediate for the regulation of organismal growth by sugars.

Sugars are not only nutrients but are also signaling molecules in plants [54,55,56]. They could act on TOR activity through new or already identified sugar signaling routes, which include hexokinase, Trehalose-6 Phosphate (Tre6P) or the production of ATP in mitochondria (Figure 1). In animal cells hexokinase II binds to mTORC1 upon glucose starvation to inhibit its downstream signaling and thus promote autophagy [57]. In plants, it was also shown that hexokinase participates in the sensing of glucose [58]. Another important signaling route involves the production of Tre6P that signals sucrose levels and inhibits the activity of SnRK1, possibly by repressing the activation of this kinase by upstream SNAK kinases [59]. Inhibition of SnRK1 would thus result in TOR activation in response to sucrose and Tre6P. Similarly, the rise in ATP levels triggered by glycolysis and mitochondrial respiration could result in an increase of TOR activity through the activation of the ATPase Reptin/Pontin complex as described above [19]. Xiong et al. [60] suggested that glucose activates TOR through glycolysis and mitochondria but independently from hexokinase or hormone signaling. However, in the *Arabidopsis* sugar-starved cell, the simultaneous addition of sucrose and of the auxin antagonist PEO-IAA repressed the induction of TOR activity as measured by S6K phosphorylation [21]. Finally, it is known in animals that the Rag GTPases pathway is involved in signaling glucose availability (and amino acid, see below) to mTORC1 by recruiting and activating the TORC1 complex at the lysosomal surface [5,9].

The relative contribution of these signaling elements to the sugar-dependent TOR activation in plants and algae remains to be precisely determined. These different signaling pathways could act independently but it is plausible that they act together and in a coordinated way to strengthen and stabilize the TOR activation signal, or in some instances to adapt sugar induction to the needs of specific tissues. Indeed, the activation of TOR by sugars may have different targets and roles in source leaves exporting sucrose or in sink tissues like roots or actively dividing meristems. The former are autotrophic for C whereas the latter need exogenous sugars for metabolism, cell division and growth. As mentioned earlier, TOR controls the communication between plant cells via plasmodesmata (PD), which are involved in the transport of macromolecules and nutrients [22]. Coherently, sugar-activated TOR seems to repress the functioning of PD to allow active phloem loading of sucrose in the phloem of photosynthetic source leaves against the gradient of sugar concentration. This process will allow sucrose transport from source to sink tissues. Conversely, TOR is less active in young sink leaves to allow a free circulation of sugars through active PD. The previous observation that phloem transport is faster in *lst8* mutants is in favor of this model [27].

In plants, the meristems are structured cell proliferation zones that persist through the life of plants and produce new organs. The activation of TOR seems to be a major effector of the stimulation of both shoot and root meristems by sugars [60,61,62,63]. In sugar-fed *Arabidopsis* roots TOR phosphorylates the transcription factor E2F, which activates cell cycling [60]. The WUSCHEL protein is needed to activate and maintain the stem cell niche in the shoot apical meristem and its expression is induced by light but also by sugars in the dark [61]. These two pathways of meristem activation are not completely similar but both seem to require TOR activity since WUSCHEL expression is no longer light- or sugar-inducible after treatment with TOR inhibitors. Growth in the apical shoot meristems is stopped in the dark but cell proliferation can be reactivated by exogenous sugars in a TOR-dependent manner [61,64]. Similarly, sugar induces callus proliferation through the activation of TOR [65]. The ErbB-3 Binding Protein 1 (EBP1) is a regulator of cell proliferation in meristems that is to some extent controlled by TOR in *Arabidopsis* [66,67]. A recent paper analyzed in more depth the mechanism by which sugars control EBP1 of the EBP1 control by TOR and demonstrated that EBP1 is induced by sugar partly through the activation of TOR [68]. This sugar–TOR regulatory axis seems to control other important biological outputs. Indeed, it was recently shown that glucose activated TOR regulates the circadian clock by shortening its period [69] and the alternative splicing of the splicing factor AtRS31 [70].

TOR also regulates the expression of many genes involved in sugar metabolism as exemplified by the transcriptomic analysis of *Arabidopsis* treated by TOR inhibitors [38,71], silenced for TOR expression [42,60,67,72] or of TORC1 mutants [27,30,32]. Conditional silencing of the *Arabidopsis* TOR gene generally leads to an accumulation of soluble sugars, amino acids, tri-acyl glycerol (TAG) and starch [67,71,72]. Similarly, inhibition of TOR in algae results in higher levels of TAG and starch, which are molecules of interest for the production of biofuels [39,73] (and see [11,12] for reviews). Interestingly, beta-amylase, which is involved in starch degradation, was identified as a TOR substrate [21].

In summary the stimulation of TOR by sugars seems to be a major signaling channel for the regulation of a vast array of diverse biological processes. It is plausible that sugars were among the primeval nutrients used by the first living cells, but also ancient signaling molecules that can convey information through TOR activation allowing cell growth and division. During the evolution of multicellular plants and animals, and with the appearance of developmental transitions and connected organs, this simple primordial regulatory loop could have been enriched by building connections to hormonal and tissue-specific signaling pathways.

## 5. Nitrogen Regulations of TORC1

A wealth of data on the regulations of TORC1 activity by inorganic and organic nitrogen sources is already available in yeast or animals [74,75,76,77]. For example in yeast TOR controls the transcriptional response to the nitrogen (N) source, and the activity of TOR is modulated by the quality and quantity of N supply [74,75]. However, despite the important roles of N nutrients and of their metabolism in plants, much less is known in photosynthetic organisms [15] (Figure 1).

In plants, N is an essential macroelement. Different indispensable molecules, such as nucleic acids, proteins, phytohormones and some components of the cellular wall, contain N that is therefore a limiting factor for plant growth [78,79]. Deprost et al. [67] studied the effect of TOR expression on root growth in the function of the nitrate environment. At standard nitrate concentration, root and shoot growth were positively correlated to the level of TOR expression in silenced or overexpressing *Arabidopsis* plants. Moreover at high nitrate concentrations, plants overexpressing TOR also displayed longer roots compared to wild-type plants. Since at the same time TOR overexpression conferred resistance to high KCl concentration, it was concluded that TOR is involved in resistance to osmotic stress.

In higher plants, N is taken by the root system and assimilated in the shoot and/or the root depending on the species. Different enzymes are involved in this process: Nitrate (NR) and Nitrite (NiR) Reductases, Glutamine Synthetase (GS) and Glutamine Oxoglutarate Aminotransferase (GOGAT) [79,80]. N assimilation is important for the plant, but it is also expensive in energy and therefore subject to regulations at different levels [81].

In yeast cells, TORC1 regulates the activity of the PP2A phosphatase complexes by controlling their association with the inhibitory beta-type subunit TAP42. The inactivation of TOR by N starvation or poor-quality sources triggers the disassociation of TAP42, the dephosphorylation of sequestering proteins and the subsequent release of transcription factors activating N assimilation [74,75,82]. TAP46 is the plant ortholog of the yeast TAP42 protein and a component of the PP2A complex that has also been shown to be a target of TOR in plants. Overexpression of TAP46 led to increased activity of NR and NiR [83], demonstrating the implication of TAP46 and TORC1 in the regulation of plant NR and NiR activities. Moreover, mutation in *LST8* increased NR and NiR, but repressed GS activities in response to long days when compared to the wild type controls [27]. Conversely, in *Chlamydomonas* TOR inhibition led to higher GS and GOGAT activities [84]. These authors also studied the link between TOR, N uptake and assimilation in *Chlamydomonas* by supplying a ^15^N labeled N source [84]. This allowed one to estimate the contribution of external N to the accumulation of amino acids observed after TOR inhibition, and which origin was debated. ^15^N incorporation into amino acids was found to be higher in cells treated by rapamycin. Therefore, a higher N assimilation seems to be the cause for the build-up of amino acids. The authors also found that ammonium uptake was higher after TOR inhibition by rapamycin. Furthermore, a phosphoproteomic study revealed that TOR activity is repressed by N starvation in *Chlamydomonas*, which may explain why N starvation induces the accumulation of TAG in algae [73,85]. Similarly, a recent study highlighted a link between low N conditions, auxin, TOR and root elongation in maize [86]. The authors suggested that low N conditions trigger an increase of shoot to root auxin transport, resulting in its accumulation in the root tip and in the upregulation of the TOR pathway. As a consequence, cell proliferation and root growth are increased, which allow the root to forage for more N in the soil.

In conclusion, it appears that in N autotrophic organisms like plants, algae and yeasts, the TORC1 complex is involved in the regulation of N metabolism. Inorganic N sources like nitrate or ammonium are first assimilated in Gln and Glu, which are subsequently used as organic N donors by transaminases for the synthesis of most other amino acids [87,88,89]. As stated above, a genetic or pharmacologic inhibition of TOR leads to the accumulation of amino acids like Gln and Asn, which are typically used for transport and storage of N in photosynthetic organisms [27,30,32,38,71,90]. In *Chlamydomonas* this amino acid accumulation is dampened by C limitation [84]. Interestingly, mutations in the YAK1 kinase, which partially suppress the decreased growth of *lst8* mutants, also strongly reduce Gln accumulation [32]. This amino acid accumulation observed after TOR inhibition could be the result of a decreased level of mRNA translation [91,92] or of an increase in autophagy, which degrades proteins [11,30,49,93]. However, as described above, Mubeen et al. [84] showed that this increase in amino acid is, for some part, the result of an increased assimilation of exogenous N since amino acid accumulation was reduced when cells were starved for nutrients and was insensitive to either inhibitors of translation or proteolysis [84].

Little information is available about the sensing of amino acids in plants [89,94], although they are known to be potent inducers of TOR in yeast and animals [10,77]. However, recently several studies reported regulations of TORC1 by amino acids in photosynthetic organisms. O’Leary et al. [95] demonstrated that Ile or Gln activate TOR in mature leaves of *Arabidopsis*. Activated TOR then diminishes night respiration and stimulates plant growth by promoting protein synthesis. Furthermore, two serendipitous approaches showed that in plants, like in other eukaryotes, branched chain amino acids activate the TOR kinase. First, it was already known that inhibition of TOR suppresses the *lrx1* (leucine-rich repeat extensin 1) mutation, which affects the development of root hairs [96]. Another suppressor of *lrx1* was recently identified as isopropylmalate synthase 1 (IPMS1), an enzyme involved in Leu biosynthesis, mutation of which resulted in the accumulation of Val and to a reduced sensitivity towards TOR inhibitors like AZD8055 [97]. In another study, the causal mutation of the *Arabidopsis eva1* mutant (ER, vacuole and actin 1) affected in vacuole morphogenesis, was also identified as an Asp/Asn change in the IPMS1 sequence [98]. As in the previous study this resulted in the build-up of Val and other branched-chain amino acids (BCAA) and in TOR activation. Accordingly, TOR inhibitors also suppressed the consequences of the *eva1* mutation on vacuolar morphogenesis. Therefore, the stimulation of TOR activity by amino acid seems to be a conserved hallmark of this kinase in eukaryotic organisms. In animals and yeast, TORC1 is recruited to the lysosomal/vacuolar surface by activated RagGTPases and the RagulaTOR complex [8,10,77]. Given the recent results, which now provide clear and independent evidence for the activation of plant TOR by BCAA, it can be anticipated that a similar mechanism may be operating in plants. However, this should now be demonstrated in future studies.

## 6. TOR Sulfur, Phosphate and Potassium

The first studies on plant nutrition showed that they need a source of C and N to grow and develop, but also of other macronutrients like sulfur, phosphate and potassium. Therefore, a consequence of one nutrient being in limited amount is a reduction of growth, but also of the assimilation of other nutrients [4]. The TOR pathway is well positioned to integrate the different nutrient availabilities signals, and to regulate the nutrient-specific metabolic arms.

In yeast, TOR regulates potassium homeostasis through the control of the plasma membrane proton pump [99], and the proton influx into the cells that is coupled to nutrient uptake was identified as a TORC1 activation signal [100]. The control of the potassium uptake by TOR was recently illustrated in *Arabidopsis* where it was shown that inhibition of TOR reduces potassium uptake in a TAP46-dependent manner [101].

In *Chlamydomonas*, an original study showed that P starvation induced autophagy and decreased TOR activity, which was measured by following RPS6 phosphorylation [102]. This drop in TOR activity seems to be linked to a decrease in LST8 protein abundance caused by P limitation. Therefore, P availability also regulates the TORC1 signaling pathway and the PSR1 (Pi Starvation Response 1) transcription factor was shown to be involved in this process. In an attempt to identify new components of the TOR signaling pathway in *Chlamydomonas*, Couso et al. [103] performed a screen for hypersensitivity to rapamycin. By this approach, they identified sensitive mutants carrying defects in VIP1 a conserved inositol hexakisphosphate (InsP6) kinase that produces InsP7 and InsP8. These mutants displayed an accumulation of storage lipids, a hallmark of the response of algae to nutrient starvation. Interestingly, VIP1 was also identified as a TOR substrate in *Arabidopsis* [21]. These findings uncover an interaction between TOR, InsP signaling and the storage of carbon reserves.

Like for the other plant macronutrient, and as expected to maintain cellular homeostasis, S availability also regulates the TOR signaling pathway. Indeed, S is an essential macronutrient for plants, which is taken up from the soil as sulfate and is needed for the synthesis of sulfur-containing molecules like Cys, Met or glucosinolates, defense-linked compounds. Inorganic S is ultimately incorporated in Cys that is used to synthesize the other S-containing molecules [104]. Recent data have provided good evidence that the availability of S is sensed through the activity of sulfite reductase (SiR), a chloroplastic enzyme reducing sulfite into sulfide [105]. Indeed, *sir* mutants have diminished levels of glucose/sucrose and consistently of TOR activity. Glucosinolates are S-containing defense metabolites derived from amino acids that, after conversion upon cell damage, give rise to an array of active compounds against pests and attackers. Malinovsky et al. [106] found that 3-hydroxypropylglucosinolate (3-OHPGSL) inhibits root development in *Arabidopsis*, but also in other plants. Interestingly this glucosinolate also affected yeast growth, which can explain why these compounds protect plants against various pests, and overexpression of TOR activity or mutations in the *Raptor2/5g* gene interfered with *Arabidopsis* responses toward 3-OHPGSL. Therefore, the biological effects of this class of compounds could be partly explained by an inhibition of the conserved TOR signaling pathway.

## 7. Conclusions and Future Prospects

The analysis of the recent findings on the cross-talks between the TOR and nutrient signaling pathways thus clearly suggests that this kinase is a central and crucial integraTOR of trophic information that controls many facets of S, N, C, P and K metabolism. Indeed, in recent years, the plant and algal TOR fields have been blooming and have borne splendid fruits. It is now clear that TOR is activated in favorable conditions by nutrients like sugars or BCAA, and that this activation results in the stimulation of growth and anabolic metabolism. Conversely, nutrient or energy limitation activates the antagonist SnRK1 kinase that inhibits TOR to promote energy saving and nutrient remobilization. It will now be of interest to determine if the regulation triggered by other nutrients like P in *Chlamydomonas* [102] are also conserved in multicellular photosynthetic organisms. In plants, which produce new organs throughout their lives, the sensing of nutrients is a strong driver of topical organ growth and of developmental plasticity through morphological adaptations. For example, N starvation reduces the shoot and stimulates root growth [86] but this adaptation is often nutrient specific: for example P limitation results in different adaptations in root morphology than N [107]. Similarly, leaf growth allows the plants to acquire more (or less when needed) light, and thus energy, and to avoid shade.

In the future, it will be uttermost interesting to determine if and how TOR is involved in these adaptive growth patterns, and genetic screens will be invaluable tools to gain further knowledge. Interestingly, a very recent report showed that the sea anemone *Nematostella vectensis* also produces new tentacles throughout its life cycle in a nutrient-regulated manner and under the control of TOR [108]. This could suggest a convergent evolution during the independent acquisition of multicellularity by plants and early animals, which rewired the primordial TOR signaling pathway to promote organ inception in a nutrient-driven manner.

The molecular determinants involved in the activation of TORC1 by amino acids, like Rag GTPases and the LAMTOR/Ragulator complex start to be quite well understood in animal and yeast cells [10,77,109]. On the contrary, the mechanisms by which sugars activate TORC1 are less clear. The molecular actors of the TOR-linked nutrient signaling pathways will have to be better identified in plants and algae. This goal could be better achieved due to a wider use of genetic screens and other genetic approaches, like genome wide association mapping, which could target the TOR signaling pathway and activity. Similarly, the control by TOR of nutrient and energy metabolism need to be better understood in photosynthetic organisms. Combinatory and integrated multi-omics analyses may help in further unveiling the entangled relations between TOR and metabolism. One difficulty is that TOR is both controlled by nutrients and a controller of many metabolic pathways utilizing these nutrients. These finely tuned regulations and equilibriums with antagonistic kinases are clearly needed to maintain homeostatic regulations of nutrients within the cell. Indeed, deregulation of TOR activity levels has a profound impact on the accumulation of either C or N compounds (for examples see [27,29,30,71,72,97,110]).

Finally, it is clear that a better understanding of the precise mechanisms linking nutrient assimilation or metabolism and plant growth, through the ancient TOR signaling pathway, would certainly help in the design of more nutrient-efficient and stress-resilient crops.
